# Genome Patterns of Selection and Introgression of Haplotypes in Natural Populations of the House Mouse (*Mus musculus*)

**DOI:** 10.1371/journal.pgen.1002891

**Published:** 2012-08-30

**Authors:** Fabian Staubach, Anna Lorenc, Philipp W. Messer, Kun Tang, Dmitri A. Petrov, Diethard Tautz

**Affiliations:** 1 Max Planck Institute for Evolutionary Biology, Plön, Germany; 2 Department of Biology, Stanford University, Stanford, California, United States of America; 3 CAS-MPG Partner Institute and Key Laboratory for Computational Biology, Shanghai Institutes for Biological Sciences, Chinese Academy of Sciences, Shanghai, China; Rice University, United States of America

## Abstract

General parameters of selection, such as the frequency and strength of positive selection in natural populations or the role of introgression, are still insufficiently understood. The house mouse (*Mus musculus*) is a particularly well-suited model system to approach such questions, since it has a defined history of splits into subspecies and populations and since extensive genome information is available. We have used high-density single-nucleotide polymorphism (SNP) typing arrays to assess genomic patterns of positive selection and introgression of alleles in two natural populations of each of the subspecies *M. m. domesticus* and *M. m. musculus*. Applying different statistical procedures, we find a large number of regions subject to apparent selective sweeps, indicating frequent positive selection on rare alleles or novel mutations. Genes in the regions include well-studied imprinted loci (e.g. Plagl1/Zac1), homologues of human genes involved in adaptations (e.g. alpha-amylase genes) or in genetic diseases (e.g. Huntingtin and Parkin). Haplotype matching between the two subspecies reveals a large number of haplotypes that show patterns of introgression from specific populations of the respective other subspecies, with at least 10% of the genome being affected by partial or full introgression. Using neutral simulations for comparison, we find that the size and the fraction of introgressed haplotypes are not compatible with a pure migration or incomplete lineage sorting model. Hence, it appears that introgressed haplotypes can rise in frequency due to positive selection and thus can contribute to the adaptive genomic landscape of natural populations. Our data support the notion that natural genomes are subject to complex adaptive processes, including the introgression of haplotypes from other differentiated populations or species at a larger scale than previously assumed for animals. This implies that some of the admixture found in inbred strains of mice may also have a natural origin.

## Author Summary

Although there is abundant evidence for phenotypic adaptation in natural populations, it is still a challenge to understand the underlying genetic processes. House mice have colonized the world in several successive waves, the most recent ones in the wake of the spread of human agriculture and trans-oceanic shipping. They have adapted to many habitats and climates, and their populations provide a rich source of opportunities for studying the impact of adaptation and positive selection on the genome. By scanning the whole genome of four natural populations of mice, we detect abundant evidence for recent positive selection, including loci that are known to be involved in genetic diseases in humans. Unexpectedly, we also find a high proportion of gene exchange between populations that have long been separated. This finding supports the notion that hybridization and transfer of alleles can significantly contribute to new genetic material subject to positive selection.

## Introduction

Genomic approaches allow an increasingly deeper insight into the forces that shape the evolution of genomes in populations. While it is evident that genome evolution depends on a balance of mutation, neutral evolution, negative selection and adaptation, the relative importance of each of these parameters is still only partly understood. Systematic genome scans for selective sweeps have shown that loci under recent positive selection can be readily detected in natural populations, but also that sweep signatures may be generated through drift effects associated with population bottlenecks or other demographic factors [1], [2]. Therefore a number of more refined statistical procedures have now been developed that allow better distinguishing positive selection from drift effects [3]–[6]. In combination with high-density genome data, it is thus possible to get deeper insights in the impact of positive selection on genome evolution.

Another factor of increasing relevance for understanding the genetic composition of natural populations is allelic introgression from other subspecies or closely related species. While it is well known that hybridization between differentiated populations and species is relevant for the evolution of many plant species, the realization that similar processes are ongoing in animal populations has only come recently [7]–[10]. While several examples of hybrid speciation in animals have now been described, there are still only few examples of introgression of specific chromosomal regions [11]–[14]. Evidence that introgressed chromosomal regions play a role in adaptation is still anecdotal and involves strong selection as in the case of warfarin resistance in mice [13] or direct mate choice signals in butterflies [14].

We use natural populations of house mice (*Mus musculus*) to study the genetics of adaptations [15]–[17]. Originating in Asia, several species and subspecies of mice have evolved within the genus *Mus* in the past million years [18]–. *M. m. domesticus* and *M. m. musculus* have separated about 300,000–500,000 years ago, which reflects in numbers of generations and relative molecular divergence the split of chimpanzees and humans. However, they are still considered subspecies, since they can be crossed and do not live in sympatry. On the other hand, hybrid males are often sterile [21], [22], leading some authors to consider them as separate species [19].

House mice have spread across the world and live in diverse habitats. As commensals of humans, they have also spread in the context of developing human agriculture in Europe and Asia within the past few thousand years, followed by a colonization of the rest of the world in the wake of transcontinental shipping within the past few hundred years [18].

**Figure 1:**
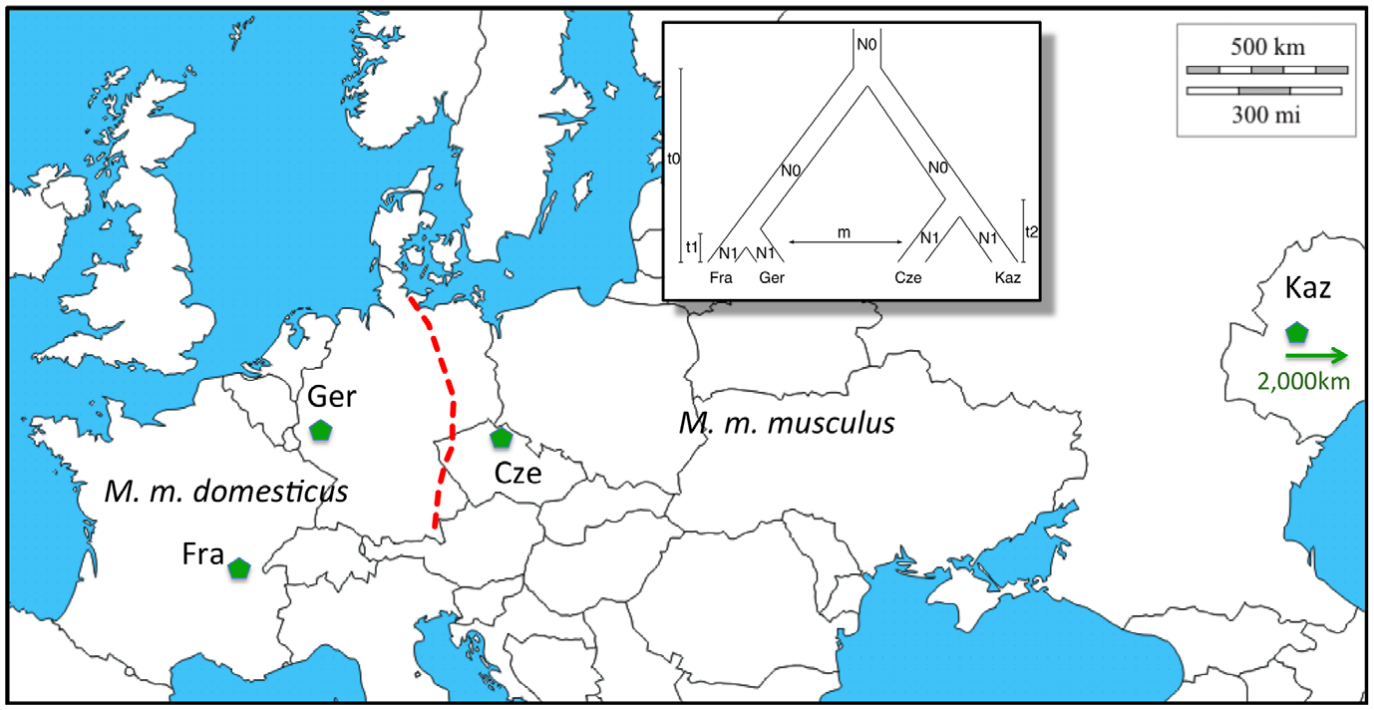
Distribution of populations and subspecies sampled. *M. m. domesticus* occurs in Western Europe, *M. m. musculus* in Eastern Europe and Asia. The dashed red line marks the contact region where a classical hybrid zone has formed. Samples come from France (Fra – region Massif Central), Germany (Ger – region Cologne/Bonn), Czech Republic (Cze – region Studenec) and Kazhakstan (Kaz – region Almaty, note that this is not on the map anymore, as indicated by the arrow). The inset depicts the phylogenetic relationship between the populations, as well as an indication of the parameters used for population modeling. Map picture based on a map from http://d-maps.com.

For the present study, we use two natural populations each from *M. m. domesticus* and *M. m. musculus* (Figure 1). The two *M. m. domesticus* populations are from Southern France (Fra) and Western Germany (Ger) and are derived from a colonization wave of Western Europe starting about 3,000 years ago [23]. The demographic history of the two *M. m. musculus* populations from the Czech Republic (Cze) and Kazakhstan (Kaz) is older but lies most likely still within the time frame of agricultural expansion of humans [24].

We use the Affymetrix Mouse Diversity Genotyping Array [25] for genotyping wild caught individuals from the respective populations. This array was designed to cover the variation for *M. m. domesticus* and *M. m. musculus* since they are the main subspecies relevant for laboratory strains of house mice. However, we found in our analysis that calling SNPs in *M. m. musculus* appears less reliable and we have therefore refined the statistical approaches to retrieve reliable genotype calls. We use these data to apply a number of statistical procedures for detecting positive selection whereby each procedure has its merits and problems for such screens. Our data from a systematic, genome wide screen for introgressed regions provide a major new insight into the frequency of introgression of chromosomal regions between specific populations. To test whether the observed frequencies of introgressed regions within a population as well as their lengths can be explained by neutral processes alone, we compare our results to coalescent simulations testing different migration parameters and suggest that both, selective sweeps caused by rare alleles or new mutations, as well as adaptive introgression of haplotypes shape the composition of the genomes of mice.

## Results

### Data quality and filtering

The Affymetrix Mouse Diversity Genotyping Array (MDGA) was designed on the basis of SNP differences between several laboratory mouse strains, both traditional and wild-derived inbred strains [25]. However, we noticed that the application of the recommended genotype calling methods to the wild mouse samples yielded an unexpectedly high fraction of SNPs incorrectly genotyped as heterozygous (see Methods). The set of SNPs interrogated by the MGDA is biased towards SNPs present in *M. m. domesticus*, although a number of SNPs polymorphic outside of this subspecies were added. Nevertheless, for samples with increasing genetic distance to *M. m. domesticus*, we saw an increase in apparently false heterozygote calls. We applied therefore the additional filtering steps described in the methods section. This yielded a list of approx. 470,000 high quality SNPs, 264,000 of which were polymorphic in *M. m. domesticus* and 167,000 were polymorphic in *M. m. musculus*. This difference reflects the ascertainment bias towards *M. m. domesticus*, since the *M. m. musculus* populations are not less polymorphic in unbiased analyses [19], [26]. We obtained high quality data from 11 unrelated wild caught individuals per population, representing 22 autosomal chromosome samples. We used males and females, implying that the coverage for the sex chromosomes was lower. Since statistical power drops with the number of chromosomes, we refrain from using data from the sex chromosomes in the further analysis.

**Figure 2:**
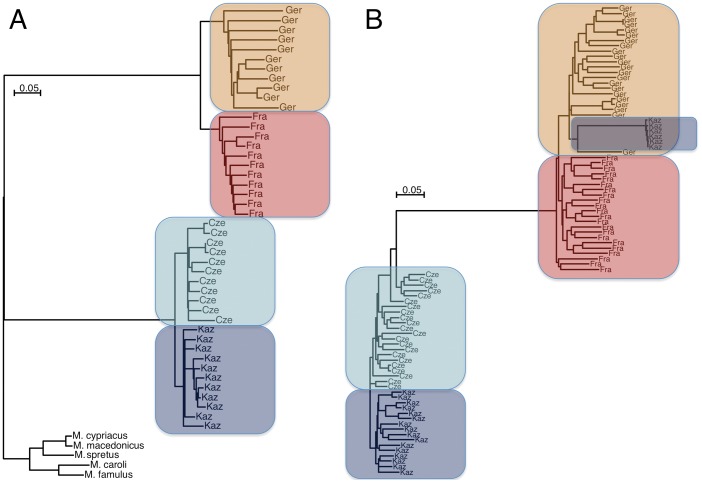
Neighbor joining trees of population samples, based on Manhattan distances. The scale bars refer to the number of substitutions per SNP genotyped. (A) For all autosomal SNPs in the study, grouped by sample and rooted by outgroup species. The shorter branch lengths for the *M. m. musculus* populations as well as the outgroups reflect the ascertainment bias for polymorphic SNPs in these subspecies and species. (B) For all haplotypes of a 6MB region on chromosome 6. Some haplotypes of Kaz group with *M. m. domesticus* due to introgression. The Cze population is partially introgressed by shorter fragments of *M. m. domesticus* haplotypes in this region (see Figure S1 for depiction of haplotype tracks of this region), which results in some paraphyletic groupings for some haplotypes.

The overall distance analysis shows that the populations and the individuals within the populations are mostly well separated, i.e. represent unrelated animals as expected based on the sampling scheme applied (Figure 2A). The distance plot shows the longest branch lengths for the *M. m. domesticus* populations, in line with the somewhat biased representation of SNPs.

### Linkage disequilibrium

**Figure 3:**
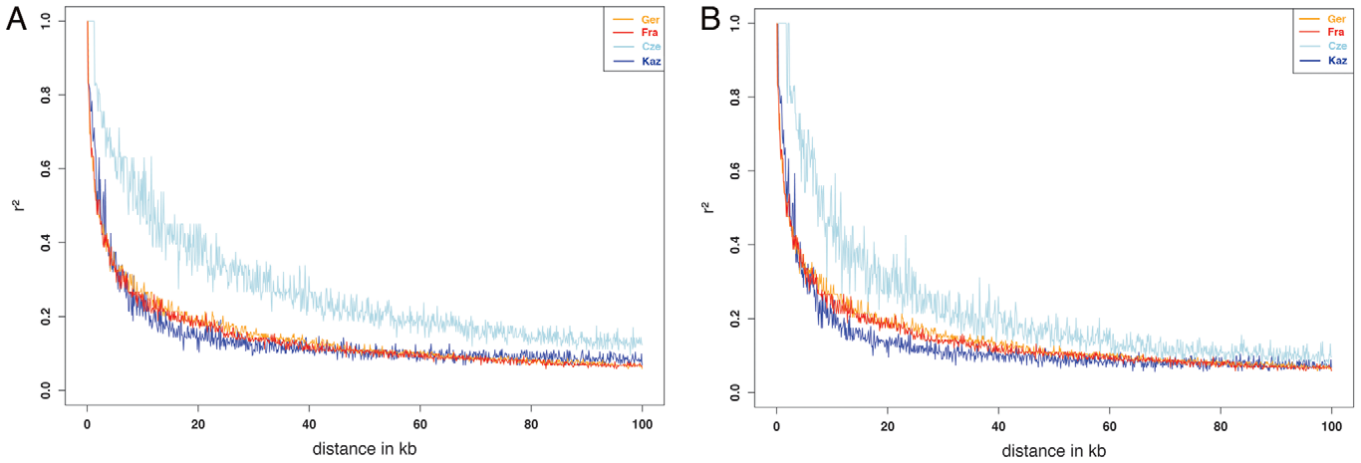
Pairwise LD (r^2^) between markers over distance for each of the populations in the study. (A) full dataset, (B) all regions of introgression removed.

Assessing linkage disequilibrium allows estimating average sizes of haplotypes that could become subject to selection in a population. Linkage disequilibrium was therefore calculated for all four populations. Figure 3A shows plots of r^2^ against physical distance for up to 100 kb. The strongest decay is seen in the *M. m. musculus* population from Kaz. This is in line with the observation that the Kaz population has a higher genetic diversity [15], most likely since it represents an ancestral population close to the source of the species. The LD decay observed for the *M. m. domesticus* populations is similar to LD estimates for a wild Arizona *M. m. domesticus* population [27] i.e. the genetic diversity is comparable between the newly colonized North American continent and the Western European populations, from which the North American mice are derived. This suggests that no severe bottleneck has occurred during the colonization of North America, probably due to multiple introgressions from various European populations.

However, the *M. m. musculus* population from the Czech Republic shows a slower decay than the Kaz population. We ascribe this in part to the presence of large introgressed regions from the other subspecies in this population (see below) and have therefore produced a second version of the LD plot excluding all regions showing signs of introgression for all populations (Figure 3B). This results indeed in a closer correspondence of the values to those of the other populations. Generally, however, linkage of SNPs beyond 100 kb is rare, even in the Cze population.

### Selective sweep scans

We used the XP-CLR statistic that evaluates allele frequency differentiation between populations [5] to identify candidate regions for selective sweeps along the chromosomes in the two population pairs. This statistic is particularly robust to ascertainment bias and population demography [5], but may still not capture all regions of interest. We have therefore applied three additional tests, Rsb, ΔDAF and F_st_. Rsb [4] evaluates the ratio of extended haplotype heterozygosities between two populations. ΔDAF [3] assesses the difference of the derived allele frequency between the two populations of interest. Derived allele states were determined from the outgroup comparisons. F_st_ is the standard measure for differentiation between populations. These three statistics were performed as sliding window analyses. Cutoff values for identifying candidate regions were determined through comparisons from simulations (see methods). Simulation results for the different parameters and lists of candidate regions are provided in Table S1.

**Figure 4:**
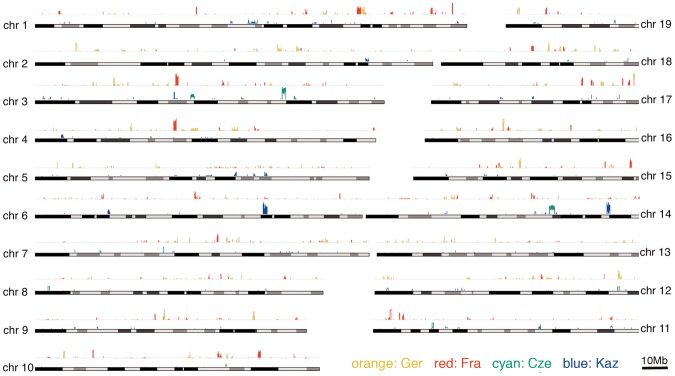
Distribution of selective sweep signatures as determined by the XP-CLR statistic for the two population comparisons across all autosomes. The figure was generated with the Genome Graphs utility of the UCSC Genome Browser [59] with custom supplied tracks that represent the XP-CLR values along the chromosomes for each population. Maximum peak sizes are up to 12, but only peaks above the simulation-derived significance cutoff at 3.5 are plotted.

The XP-CLR statistic was used to visualize the distribution of potential sweep regions across the chromosomes (Figure 4). All regions above a threshold of 3.5 can be considered significant based on comparisons with simulated data (see methods). As an additional criterion to identify a credible region, we required it to have at least half of 12 consecutive grid segments (5 kb for *M. m. domesticus*, 20 kb for *M. m. musculus* - see methods) to have a value above 3.5. These candidate regions are listed in Table S1.

Table 1 lists summaries on the number of significant chromosome regions detected for each of the statistics at the chosen cutoff levels based on comparisons with neutral simulations (see methods). It also provides the overlap of these regions with regions identified by the respective other statistics for the individual populations. The results show that the different statistical measures overlap only partially, indicating that each of them is indeed measuring different aspects of a complex pattern.

**Table 1:** Summary for sweep statistics.

	Germany (Ger)	France (Fra)	Czech Republic (Cze)	Kazhakstan (Kaz)
XP-CLR^1^ (N)	287	254	35	73
fraction of genome	0.87%	0.82%	0.65%	0.82%
average size (bp)	79,007	84,350	488,571	293,151
maximum size (bp)	1,430,000	1,335,000	2,460,000	1,940,000
deltaDAF^2^ (N)	32	9	106	16
Rsb^3^ (N)	687	764	1054	217
XP-CLR+Rsb (N)	64	87	10	7
deltDAF+Rsb (N)	12	0	43	2
XP-CLR+deltDAF (N)	5	1	3	1
XP-CLR+deltDAF+Rsb (N)	5	0	3	1
F_st_ 4 (N)	369		414	

1 Cross-population composite likelihood ratio test [5].

2 Difference in derived allele frequency [6].

3 Ratio of integrated extended haplotypes between populations [4].

4 Fixation index in 100 kb windows.

N = number of significant regions detected for the respective statistics.

### Example of loci under recent positive selection

Table 2 lists regions and genes that were found by a combination of at least two statistics (XP-CLR and ΔDAF), but mostly by all four. They differ greatly in size (from approx. 150 kb to 2,390 kb) and gene content (from zero to 35). Several of them include genes known to be involved in causing genetic diseases in humans, such as the locus involved in Parkinson's disease (region Ger3, Park2 [28]), or juvenile polyopsis (region Ger2, Bmpr1a [29]). Ger2 includes also the locus for Melanopsin (Opn4), a gene involved in circadian phase shifting [30]. The genes in one region are known to be involved in imprinting in human and mouse placenta (region Ger1 - Zac/Plagl1 [31], [32]) and include a further gene suspected to be a risk factor in Parkinson's disease (Phactr2 [33]). Another region has been described to be adaptive in humans (Cze1, amylase1 [34]). One region includes a gene involved in auditory and behavioral functions in mice (region Kaz1, Slitrk6 [35]). Two regions contain no known genes (Fra1 and Cze3) but include several highly constrained regions, according to GERP scores (implemented in UCSC genome browser [36]). Using gene ontology (GO) analysis on each of the gene lists obtained from the different statistics, we did not find consistent patterns of gene classes to be preferentially affected. Most lists yielded no significant enrichment; other lists yielded enrichments that were specific to the list and population (Table S2). Hence, it appears that the full spectrum of genes can potentially become subject to positive selection.

**Table 2:** Examples of selective sweep regions identified by multiple statistics.

	Location^2^	size (kb)	classification	genes in the region^1^
Ger1	chr1012719281–13144281	425	coding, imprinted, disease related	Phactr2 (phosphatase and actin regulator 2 isoform A), Plagl1 (pleiomorphic adenoma gene-like 1), Zac1 (zinc finger protein)
Ger2	chr1435,222,025–35,451,763^3^	230	coding, disease related	Bmpr1a (bone morphogenetic protein receptor type-1A), Ldb3 (LIM domain-binding protein 3 isoform), Opn4 (melanopsin isoform 1)
Ger3	chr1711,520,278–12,071,811	550	coding, disease related	Park2 (parkinson protein 2 - E3 ubiquitin-protein ligase parkin)
Fra1	chr1259,560,813–59,710,943	150	non-coding	center of 680 kb non-coding region
Fra2	chr1538,059,935–38,210,538	150	coding	Odf1, main protein of sperm tail outer dense fibers
Cze1	chr3112,689,523–115,078,986	2,390	coding, disease related	Amy2b (amylase 2b isoform 1), Amy2a5 (pancreatic alpha-amylase), Amy1 (alpha-amylase 1), Col11a1 (collagen alpha-1(XI) chain), Olfm3 (olfactomedin 3)
Cze2	chr729571436–30171436	600	coding	ca 35 partially overlapping genes
Cze3	chr86013855–6933855	920	non-coding	center of an approx 3 Mb non-coding region
Kaz1	chr14109933747–111553747	1,620	coding (mostly non-coding)	Slitrk6 (SLIT and NTRK-like protein 6)

1 Lists only genes in the region with functional information in the mouse.

2 Overlapping windows of different statistics combined.

3 Combines three neighboring XP-CLR positive regions.

### Introgression across subspecies

**Figure 5:**
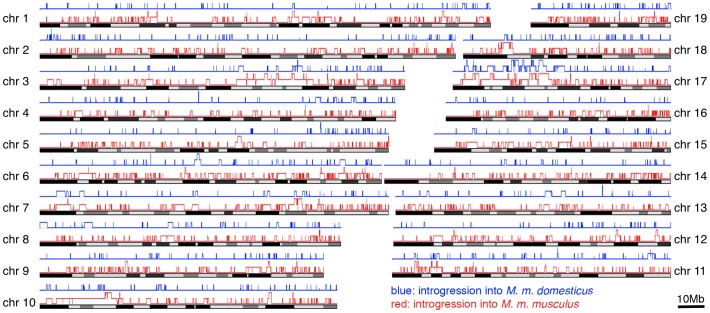
Distribution of introgressed regions across the chromosomes. Introgressed regions into *M. m. domesticus* in blue, into *M. m. musculus* in red. Elevated blocks indicate regions found in both populations of the respective subspecies. The figure was generated with the Genome Graphs utility of the UCSC Genome Browser [59].

During the analysis of our data we noticed that some haplotypes of a given population appeared to be more similar to haplotypes found in the other subspecies (an example is shown in Figure 2B). This is indicative of population-specific introgression across subspecies boundaries. To systematically detect such regions, we used the Hapmix algorithm [37]. This program assumes that one has two non-admixed populations to identify admixture in the focal population. In our framework, we use the sister population of a given subspecies as one possible source and the populations of the other subspecies as another source (e.g. if Ger is our focal population we use Fra as one potential source and combine Kaz and Cze into the other potential source). This allows us to detect population-specific introgression, but we would evidently miss all cases where introgresssion occurred into both populations of a given subspecies. Hence, our approach detects only a minimal set of introgression events. Table 3 summarizes the number and size of regions that had at least one introgressed haplotype; all regions are listed in Table S3. The fraction of introgressed haplotypes is particularly large in the Cze population, possibly due to its proximity to the hybrid zone. Both *M. m. musculus* populations have on average larger introgressed regions, with the largest of approximately 20MB in the Cze population. When all regions are considered, a total of 25% of the genome is subject to mutual introgression between the sub-species and 9.6% when the Cze population is disregarded. The regions are distributed across all chromosomes (Figure 5). The proximal half of chromosome 17 shows some clustering and overlap between the populations, which is likely to be due to the t-haplotype, a meiotic drive element consisting of several inversions reducing local recombination. The t-haplotype covers the centromeric half of chromosome 17 and has introgressed from another species [38]. Therefore it is recognized as mutually introgressed by the Hapmix algorithm in our populations. Hence, we have omitted this region from the further statistical calculations, although it has only little impact on the overall pattern.

**Table 3:** Genome regions affected by introgression.

	Ger	Fra	Cze	Kaz	all w/o Cze	all
Hapmix^1^ (N)	374	466	1,316	270	1,110	2,426
average size (bp)	189,757	150,514	347,088	461,483	239,377	297,806
maximum size (bp)	2,292,307	3,808,863	19,446,657	12,506,547	12,506,547	19,446,657
part of genome^2^ (%)	2.8	2.8	17.7	4.9	9.6	24.8
FDR (%)	2.2	1.2	3.8	5.0		

1 Region in proximal half of chromosome 17 (t-region) omitted.

2 Corrected for omitted genome region.

**Figure 6:**
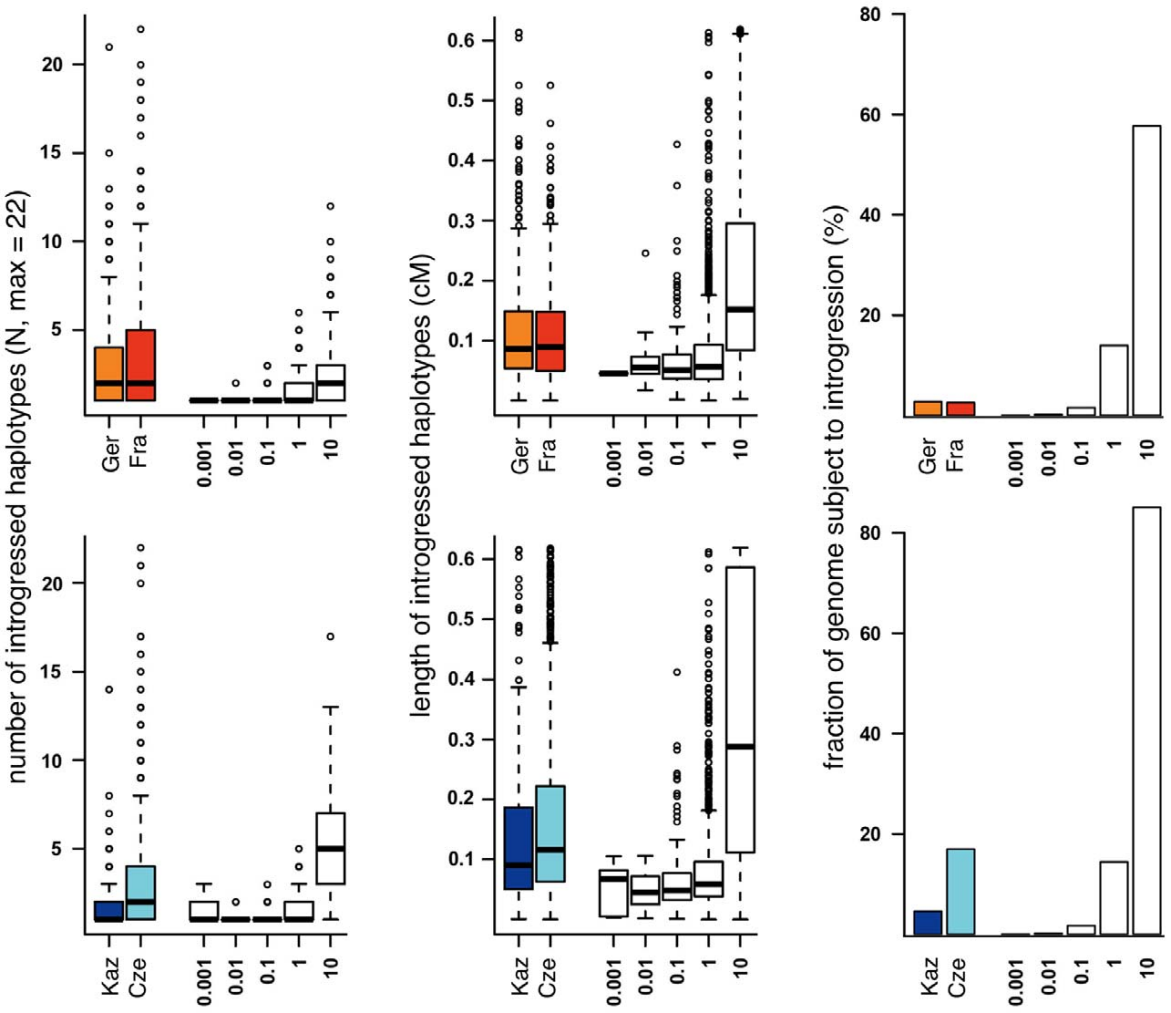
Comparison of introgression parameters (colored bars) with neutral models for introgression (white bars, numbers on the x-axis refer to migration rates in 4Nm). Top: *M. m. domesticus* populations, bottom: *M. m. musculus* populations. Thick horizontal lines represent the median, boxes range from first to third quartile, whiskers extend to 1.5 times the interquartile range, data points outside that range are drawn as circles.

To assess whether the observed introgression could be compatible with a neutral migration-introgression model, we simulated datasets with varying population size and migration rates ranging from very small migration rates, which one would expect under sporadic long distance migration model, to relatively frequent migration, such as to be expected in the proximity to the hybrid zone (see methods). We applied Hapmix with the same settings as used for analysis of the empirical data to the simulated data sets. Figure 6 suggests that the observed frequency of introgressed haplotypes (Figure 6 left) as well as observed haplotype length (Figure 6 middle) would require migration rates higher than 4Nm = 1 in a neutral model. However migration rates of that magnitude are incompatible with the observed fraction of the genome affected by migration for all but the Cze population (Figure 6 right). If migration rates were sufficiently high to generate the observed high frequencies and lengths of introgressed haplotypes, a fraction of >15% of the genome should show evidence of introgression. The observed fractions of the genome subject to introgression are significantly lower, 2.8% for the Ger and Fra populations and 5.8% for the Kaz population (Table 3). On the other hand, the relative frequency of introgressed haplotypes is higher than expected in a neutral model. At a threshold of 4Nm = 1 we would expect less than 1% of all introgressed haplotypes to be at a frequency larger than 4 out of 22, but the observed fractions of haplotypes with higher frequency are much larger (Kaz: 8.1%, Cze 19.7%, Ger 20.3% and Fra 27%). This suggests that a large fraction of the haplotypes is subject to forces that lead to a faster increase in frequency than expected by chance.

We can estimate significance of this observation by assuming a migration rate of 4Nm = 1. At this migration rate simulations match the proportion of the genome subject to introgression in the Czech population while for all other populations a greater fraction of the genome would be expected to have introgressed leaving p values more conservative. Nevertheless the observed frequency of introgressed haplotypes is significantly higher in all populations compared to neutral simulations (p<2.2e-16 for Germany, France and Czech Republic and p = 0.02 for Kazakhstan).

The introgressed regions could also reflect incomplete lineage sorting since the split of the sub-species, as it has been proposed in the analysis of several specific loci [19]. We have therefore sought to estimate this effect within our data and analysis framework. Using Hapmix on simulated data without migration, we find only a small number of positive regions per population, which allows calculating a false discovery rate (FDR) of 5% or less (Table 3).

GO analysis of genes covered by introgressed regions (Table S3) showed some enrichment terms due to the inclusion of gene clusters. Most notably, a large olfactory receptor gene cluster on chromosome 7 (see below), the bitter taste receptor gene cluster on chromosome 6 and the cluster of epidermis genes Sprr and Lce on chromosome 3 show complex mutual patterns of introgression.

### Visualization

**Figure 7:**
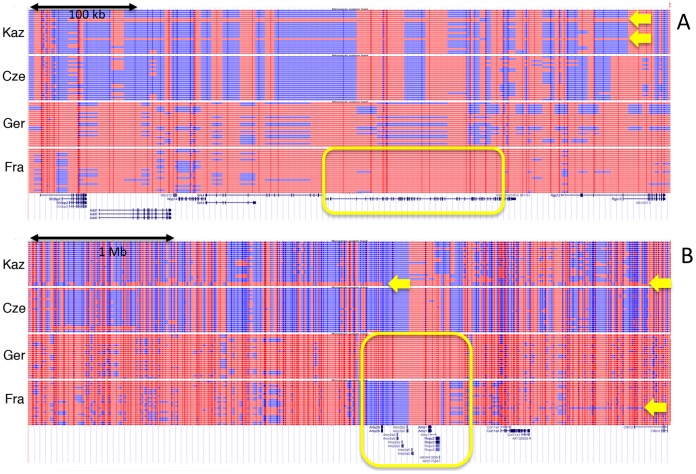
Haplotype tracks showing sweeps and introgression regions. Lines represent two reconstructed haplotypes for each individual. The data are displayed as custom tracks in the UCSC browser [59]. SNP positions are depicted as vertical bars, SNP variants that are more frequent in *M. m. domesticus* are in red, *M. m. musculus* in blue. Spaces between the SNP positions are filled with the color corresponding to the flanking SNPs. If these are of different color, the space is broken up in the middle. Known genes in the regions are depicted below (taken from the UCSC Genome Browser database - [14]). Sweep regions and corresponding genes are highlighted as yellow boxes, introgressed haplotypes are indicated by yellow arrows that point to the respective haplotype tracks that include them. (A) Genome region around the Huntingtin (Htt) locus on chromosome 5, showing a sweep in the Fra population and introgression into the Kaz population. (B) Genome region around the alpha-amylase gene cluster (Amy) on chromosome 3, with mutual sweeps in the Ger and Fra population and introgression in the Kaz and Fra population. Note that the introgressed haplotype in Kaz extends further than depicted here (covering approx. 33 Mb in several pieces).

To visualize selective sweeps and introgressed regions in the population and chromosome context, we generated UCSC browser custom tracks based on phased data from the individuals in each population. Figure 7 shows two examples of such tracks with their associated genes. Figure 7A shows a sweep around the locus involved in Huntington's disease in humans (Huntingtin) in the Fra population. Interestingly, the region is at the same time part of a larger region that has introgressed into the Kaz population. Figure 7B shows the sweeps around the alpha-amylase genes (see Cze1 in Table 2). Although sweeps are evident for Fra, Ger and Cze, only the Cze region is identified by all three statistics. Again, there is a long region of introgression into Kaz and a shorter one into Fra. In fact, the sweep allele in Fra is apparently also derived from an introgressed haplotype. Additional example regions for sweeps and introgression are shown in Figure S1.

## Discussion

Our analysis provides an insight into the complex interplay between selection, introgression and drift in natural populations of the house mouse. We cannot even claim that we have identified all complexities, since the deme structure of mice with local inbreeding is difficult to capture in standard statistical models. However, our sampling scheme for the local populations was designed to get a representative sample across a whole area, which is more likely to represent a more long-term stable population sample [15]. In our previous microsatellite based screens for selective sweeps in these populations [15], [16], we had already found that patterns of positive selection can be readily detected and we estimated a frequency of more than one cycle of positive selection per 100 generations [16]. The SNP based survey here is compatible with this finding, although the results cannot be directly compared, since microsatellites have a much higher mutation rate than SNPs and will therefore detect primarily very recent events. An unexpected finding in our study here was that introgression of genomic fragments across the *M. m. domesticus*/*M. m. musculus* boundary does appear to play a larger role than previously expected and that the frequency and size distribution of the introgressed fragments can not be explained by a neutral introgression model. This suggests a general interplay between selection and introgression shaping the genomes, which is difficult to disentangle. Still, we discuss these factors in turn in the following, since we have used different approaches to detect them.

### Patterns of positive selection

A number of statistical methods have been developed to infer selection from SNP data in population surveys. The best datasets for applying such tests were so far available for human populations [1], [3], [6], [39]. The data that we obtained from the mouse arrays now begin to parallel the human data availability, at least with respect to SNP coverage. We have explored a range of reasonable demographic scenarios that are in line with previous population genetic results [19], [20], [26], [40] to obtain estimates for cutoff values reflecting neutral processes for the various statistics. Since it became clear that adaptive introgression plays a significant role in shaping polymorphisms, and since this would be difficult to model because of the many combinations of parameters that could play a role, we refrained from an even deeper exploration of the demographic parameter space. Each of the statistics yields a list of candidate regions, but the overlap between these lists is limited, as it was also observed for human data [2]. This suggests that the statistical procedures are necessarily limited to their respective model of evolution, while the reality of adaptive processes is dynamical and complex and involves also as yet unsolved questions of the influence of deme structures and long-term stability of local populations.

The XP-CLR statistic appears currently best suited to deal with this complexity (as tested by the authors [5]), although it is necessarily also limited with respect to not taking into account possible confounding influences of introgression. Under our cutoff chosen for candidate regions (which can be considered to be conservative), we find that about 0.8% of the genome are covered in each population, suggesting that genetic draft contributes significantly to the evolutionary genome dynamics in mouse populations.

Depending on effective population size and other parameters, significant sweep signatures may be traceable for only 10,000 generations [41], which is not much more than the estimated time of separation between the Fra and Ger populations. In our previous microsatellite screen we found that 200–300 selective sweeps should have occurred in each lineage since their separation [16]. This is in accordance with the number of regions identified in the XP-CLR statistic (287 for Ger and 254 for Fra), although one has to keep in mind that the exact number depends on the cutoff criteria. Still, the order of magnitude appears to correspond, although the approaches are rather different [16]. The Cze and Kaz populations may have been separated for a longer time, causing older signatures of selection to increasingly vanish and become undetectable. The fact that a smaller number of regions are identified in these populations may be due to a lower density of informative SNPs in *M. m. musculus* populations, or the population history may be more complex, i.e. the model applied for obtaining a cutoff may not be fully adequate. Still, independent of the exact number of sweep regions identified, it is evident that sweep candidates can be readily retrieved in the mouse, implying that new mutations or rare alleles can frequently become subject to positive selection. An in depth analysis of this question in humans has suggested that selective sweeps based on new mutations have been rare in the recent human history [39]. This analysis was based on re-sequencing data, which are not yet available for our mouse populations. Still, given that both the microsatellite approach [16], as well as the data here show that many classic signatures of sweeps can be identified, it would appear that mouse populations are somewhat different from human populations in this respect and possibly also more typical for natural populations.

Some of the gene regions that were identified by more than one statistic are of particular biological interest. For example, genes that have been implicated in causing diseases in humans would have been expected to be primarily under purifying selection, since most of them are derived from old conserved genes [42]. The fact that two of the most studied disease genes in humans (Huntingtin and Parkin) come out as loci that are subject to positive selection and introgression suggests that evolution keeps shaping the function of such genes. This supports the notion that medical aspects of gene functions can profit from taking evolutionary considerations into account [43].

### Introgression

It had previously been noticed that different gene regions can have different phylogenetic genealogies between populations of *M. m. domesticus* and *M. m. musculus* [19], [40], [44]. Although incomplete lineage sorting is formally another explanation for phylogenetic discordance at some loci [19], this explanation could be excluded for the highly polymorphic minisatellite loci studied by [44]. Our simulation analysis does also not suggest that incomplete lineage sorting contributes much to the observed sharing of haplotypes. Hence, we conclude that a relatively large fraction of the genome can become subject to introgression from haplotypes of the other subspecies, even between populations that are very far apart of each other. Introgression across the hybrid zone between *M. m. domesticus* and *M. m. musculus* has suggested that the median cline width of introgressed markers is about 30 km [45]. However, these authors noticed also a long tail in the distribution and some of the markers with long-range introgression fall into regions that we see as introgressed haplotypes (not shown). Our samples of the Cze population came from a region about 200 km away from the hybrid zone, the ones for the Ger population are about 450 km away (in east-west direction). In our analysis, we find the Cze sample much more invaded by *M. m. domesticus* haplotypes, which is in agreement with closer location to the hybrid zone, but also in line with previous findings that there is usually an asymmetry of introgression of markers from *M. m. domesticus* into *M. m. musculus* [45], [46]. However, the Fra and the Kaz populations are far away from a population of the respective other subspecies (Figure 1) and still show a similar extent of introgression as the populations closer to the hybrid zone. Hence, it can only be rare long-distance dispersal, most likely aided by human transport that leads to the transfer of haplotypes rather than slow diffusion emerging from the hybrid zone.

Once an introgression has occurred, the haplotypes should quickly break up through recombination while being subject to drift. The long introgressed haplotypes that we have observed are either refractory to recombination, for example due to an inversion, or confer a selective advantage, i.e. spread faster than they can break down. Although we cannot rule out hat inversions exist for some regions, the visual analysis suggests that breakdown products can segregate in parallel to the longest haplotypes (Figure S1). Hence, recombination does appear to take place during the spread of the haplotypes. We have found only few cases where a detectable haplotype was fixed and is associated with a sweep signature (Figure 7B). However, if we compare the frequency and length distribution with our simulated data, we have to conclude that a major fraction of introgressed haplotypes is subject to positive selection, although not yet fixed. Different scenarios could explain this observation. Selection coefficients might not be sufficiently strong to fix the introgressed haplotypes quickly and at the time when the relevant advantageous alleles get fixed, the haplotype may have broken down to a size where it is beyond the threshold of recognition in our data. Alternatively, frequency dependent or balancing selective forces could also play a role in preventing fixation.

Adaptive introgression of chromosome regions, even across species boundaries, has been described for alleles that cause warfarin resistance in mice [13]. Also, the phenomenon of hybrid speciation leading to new adaptations suggests that the introgression and mixing of haplotypes from different lineages can well become advantageous [7]–[10] and can quickly lead to new adaptive lineages [47]. Hybrid speciation in butterflies has been directly linked to introgressed haplotypes involved in wing patterning [14]. Hence, there is abundant evidence that genomic material coming from related species can confer an advantage to populations. Although *M. m. domesticus* and *M. m. musculus* are not formally designated as separate species, they are in a stage of divergence that is typical for many closely related species, where hybridization is still possible. Hence, we expect that the degree of introgression that we see between our subspecies may occur in a similar way among many closely related species pairs.

There is an ongoing discussion on whether inbred laboratory mouse strains are ultimately derived from a mixture of subspecies crossed in during some phase of the inbreeding process [48], [49]. However, with our finding of large-scale introgression of haplotypes under natural conditions, this question will become of less relevance, since a truly “pure” population might not exist in nature anyway. In fact, a previous study of wild derived inbred mouse strains typed with a smaller set of SNPs had already suggested a rather high degree of introgression of haplotypes between the subspecies [50]. However, the *M. m. musculus* strains analyzed in this study were derived from the Czech Republic, where a major influence of introgression from the hybrid zone occurs (as we see it also in our data). Moreover, the possible influence of adaptive introgression was not tested by these authors.

The GO analysis did provide some enrichment terms of gene classes for introgressed regions, but closer analysis showed that this is due to the fact that a few regions encode gene clusters, such as olfactory genes or bitter taste receptor genes. This leads to a relative over-representation of terms like “sensory perception” or “G-protein coupled receptor activity” for the whole statistic, although this is only due to these clusters. Overall, we see no general pattern of types of genes subject to introgression across the genome. Still, the fact that the gene clusters mentioned are involved in introgression is of particular interest, since they code for genes that are very likely subject to population specific adaptations, as has also been noted previously [45]. Other gene clusters, like the major urinary proteins (MUPs), which are involved in individual recognition [51], would thus also expected to be subject to introgression, but there are still problems with an appropriate annotation of these regions [52], i.e. they are not sufficiently covered in the SNP array and were therefore outside our detection capacity. Hence, dedicated studies will be required to explore this further.

### Conclusion

Our data suggest that the genome of natural populations is not only shaped by drift and selection, but also by introgression from sister-species. As discussed above, there is increasing evidence that this occurs also in other animal species, including even humans, where haplotypes from Neandertals and Denisovans have introgressed in some populations [12], [53]. We should therefore like to propose “comet alleles” as a generic term for such haplotypes which introgress across sub-species and species boundaries. Like real comets, which come from the outer solar system, they come from other evolutionary lineages. Like comets, which dip into the solar system, “comet alleles” dip into another evolutionary lineage, can become more shiny (i.e. increase locally in frequency), can break up (i.e. can recombine), can disappear again, or can impact (i.e. get fixed). We anticipate that comet alleles will be found frequently among closely related species, once high-density polymorphism data become more broadly available. This will posit new challenges for designing appropriate population models for the investigation of the role of positive selection in shaping the genomes.

## Materials and Methods

### Ethics statement

All animal work followed the legal requirements, was registered under number V312-72241.123-34 (97-8/07) and approved by the ethics commission of the Ministerium für Landwirtschaft, Umwelt und ländliche Räume, Kiel (Germany) on 27. 12. 2007.

### Mouse sampling and DNA extraction

Mice were collected in Germany (in the Cologne-Bonn area), France (in the Massif Central), Czech Republic (around Studenec) and Kazakhstan (around Almaty) taking care to obtain a representative local sample as described in [15]. DNA samples were run on a 0.7% agarose gel to ensure high DNA quality prior to further processing. DNA samples of eleven mice per population plus the outgroup species *Mus caroli*, *Mus famulus*, *Mus spretus*, *Mus cypriacus*, and *Mus macedonicus* were processed. SNP typing microarrays (Affymetrix Mouse Diversity Genotyping Array) were processed according to the recommended procedures of the supplier.

### Filtering SNPs to have only reliable calls

First, we excluded from the analysis all SNPs flagged as unreliable in the array annotation file provided by Affymetrix. Because we found BRLMM-P calling (Affymetrix) to be susceptible to false heterozygote calls when a probe set is not compatible with a sample (sequences complementary to the probes are missing in a genome, and this concerns mostly *M. m. musculus* samples), all SNPs called heterozygous were re-checked. Only those, which had an average intensity for both alleles higher than a cutoff, were retained. The cutoff was the first quantile (0.1) of homozygous calls in the reference (*M. m. domesticus*) samples, or when those were unavailable, prior homozygote cluster centers, distributed by Affymetrix, were used to determine the cutoffs. As this procedure still does not remove all false heterozygous calls, we conservatively excluded also from each population SNPs not concordant with Hardy-Weinberg equilibrium (one sided Fisher test with p<0.01). Protocol S1 provides additional details.

### Assessment of linkage disequilibrium

We used the command line option of haploview [62] version 4.1 with default settings on data filtered as previously described to calculate r^2^. For plotting, all pairs of SNPs with a highly similar physical distance (within a 100 bp range) were binned into groups followed by calculation of median r^2^ for each bin.

### Statistics to trace selective sweeps

Among the large number of available statistics to search for natural selection in genomic data, we chose a number of complementary statistics to suit our experimental setup. We focused our search on comparisons between sister populations from the same subspecies (Ger vs. Fra, Kaz vs. Cze) to minimize effects of ascertainment bias and demography that could arise in cross-subspecies comparisons. We found that Rsb [4] and XP-CLR [5] are well suited for comparing the sister populations while focusing on two different informative aspects of the data. XP-CLR uses population differentiation as a means to detect regions under selection and hence is particularly robust against ascertainment bias, while Rsb compares length and frequency of haplotypes between populations. We then supplemented these statistics with the difference in derived allele frequency (ΔDAF [6]) and the classical measure of population differentiation F_st_.

XP-CLR values were calculated using scripts made available by [5] at (http://genepath.med.harvard.edu/reich). The following parameters were used: window size 0.02 cm, grid size 5 kb for *M. m. domesticus* populations and 20 kb for *M. m. musculus*, maximum # of SNPs within a window 50, correlation level from which the SNPs contribution to XP-CLR result was down weighted 0.95. Genetic positions based on [54] from the Affymetrix annotation file were taken as input. For each population the sister population of the same subspecies was taken as a reference for generating XP-CLR values. To identify candidate regions for a population, we normalized its XP-CLR values by subtracting the mean and dividing by the standard deviation. Chromosomal regions where at least half of the consecutive 12 grid segments were above the simulation-based cutoff value were designated candidate regions. Overlapping regions were collapsed.

For Rsb, ΔDAF and F_st_ we used a sliding window approach (100 kb windows, 50 kb increment) to detect candidate windows outside simulation defined cutoffs. Different cutoff levels are provided in Table S1. For the analysis presented in the text, we used the most stringent one at p<0.001. Windows with fewer than 10 SNPs were discarded. Rsb values were calculated applying Perl scripts as described in [4] to phased data (fastphase [55]). Because large gaps in the available genotype information can cause misleading Rsb results, SNPs closer than 300 kb to gaps of at least 1 Mb were removed from the Rsb analysis. ΔDAF was calculated as described in [6]. We used the genotyping data from *Mus spretus*, *Mus cypriacus* and *Mus macedonicus* to determine the ancestral state of SNPs. In cases were data for these three species were either not available or inconclusive, the ancestral state was inferred from either *Mus famulus* or *Mus caroli*, if possible. Heterozygous genotypes in the outgroup species were disregarded. F_st_ was calculated using the formula F_st_ = 1-Hs/Ht where Hs is the mean heterozygosity of the two populations and Ht is twice the product of the heterozygosities within populations. We also applied the correction for sample size developed by [57] with almost identical results (data not shown) due to our filtering procedure and proceeded with the simpler formula above.

### Detection of introgressed haplotypes

Hapmix [37] was developed to infer the ancestries of chromosomal fragments of an admixed individual using genotype information from two potential source populations in a Hidden Markov Model. We leveraged this approach to detect chromosomal fragments that have introgressed from the other subspecies into our population of interest. Therefore we chose the sister population from the same subspecies as one potential source and the other subspecies as alternative source. If the ancestry of a chromosomal region was assigned to the alternative source, i.e. to the other subspecies, we regarded this region as introgressed. Adjacent and overlapping chromosomal regions in which the ancestry of at least one haplotype was assigned to the other subspecies were merged into introgressed regions that, following the principle of parsimony, stem most likely from a single introgression event. Frequencies of comet haplotypes were determined by counting the number of individuals within such a region that show evidence for introgression using an R script.

We applied Hapmix with standard settings but adjusted the recombination parameters to values observed in mouse as described in [54] to phased data (fastphase [55]). Genetic positions from the Affymetrix annotation file were taken as input for Hapmix. To avoid confounding resolution effects of the mouse linkage map on detection and assignment of start and end points of introgressed haplotypes, we allowed for an appropriate amount of recombination between markers that differed in physical position but were assigned the same genetic position on the mouse linkage map. Therefore the genetic distance between markers adjacent to a block of markers at the same genetic position was split amongst the markers in between according to physical distance.

Time since admixture was set to 100 generations and the miscopying parameter to 0.0005, which we found to allow for optimal use of the resolution of the MWGDA to also detect smaller introgressed haplotypes with reasonable power by intense visual verification. The minimum per SNP certainty threshold to call a SNP introgressed was 0.9 (default setting).

To estimate a false discovery rate (FDR) for introgressed regions we applied Hapmix with the same settings we used for empirical data to simulations without introgression (see next paragraph for demographic parameters). Because migration is set to zero in these simulations, the remaining regions that are detected as being introgressed reflect incomplete lineage sorting. Assuming a genome size of 2.7 Gb in mice FDR = 2.7*n/o, where n is the number of regions detected in 1 Gb of simulated data without migration and o is the number of observed introgressed regions in the empirical data.

### Simulations and cutoffs

To determine cutoffs for sweep statistics under a neutral scenario we employed coalescent simulations with ms [56]. We combined data from previous studies to generate a likely demographic scenario [19], [20], [23], [26], [40] using the following parameters (compare inset Figure 1 for annotation): N0 = 100,000; N1 = 10,000; 20,000; 50,000; 100,000; t0 = 1,000,000; t1 = 3,000; t2 = 10,000. We simulated 1,000 regions of 1 Mb size for each scenario. To analyze the expectation of introgressed haplotype length and frequency under a neutral model we added migration between subspecies at varying rates (4Nm = 0.001, 0.01, 0.1, 10) to the model and varied population size between 100,000 and 10,000 representing a 10-fold reduction in effective population size after the split of sister populations until present.

### Rejection sampling

To resemble the ascertainment scheme of the chip design in our neutral simulations we extend an approach first introduced by [58]. We use a rejection-sampling algorithm to ascertain a subset of SNPs from a neutral simulation such that the frequency spectra in the four subpopulations estimated for the ascertained SNP set resemble those observed in the chip data. Our algorithm achieves this by minimizing the distance
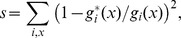
where 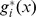 is the number of SNPs in the ascertained set for which the derived allele is at frequency *x* in subpopulation *i*, and 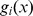 is the respective observed number in the chip data. The summation is over all four subpopulations and all population frequencies in the data, including loss (*x* = 0) and fixation (*x* = 1) in a subpopulation. We start our algorithm with an empty set of ascertained SNPs. We then choose a random SNP from the simulation data, add it to the ascertained set, and calculate the new *s*. If the new *s* is smaller than the *s* of the set without this SNP, the SNP is kept in the ascertained set, otherwise it is rejected. We then chose a random SNP from the ascertained set, remove it, and check whether this lowers *s* or not. If it does, the SNP is removed from the ascertained set, otherwise it is kept. We then choose again a random SNP from the simulation data and repeat the above process until no further improvement in *s* has been achieved over a substantial number of cycles, which we defined to be 10 times the number of segregating sites in the simulation sample.

### Other analyses

Data visualization was done with the UCSC genome browser [59] for the mouse genome assembly mm9. Custom tracks were generated for the phased haplotype data (using fastphase [55]) and the windows identified by the different statistics. The “Tables” function [60] and the “Genome Graphs” utility were used to calculate fractions of genome affected and to retrieve gene lists overlapping with the respective windows. Gene lists were then analyzed with FuncAssociate 2.0 [61] for enrichment of GO terms. The trees shown in Figure 2 are based on Manhattan distances. Because the polymorphisms typed with the WGDA are not only the function of a natural mutational process, but also of ascertainment bias, standard distance corrections like the Jukes-Cantor model are not suitable for such a data structure. Manhattan distances do not apply assumptions about underlying mutational processes.

### Data availability

The SNP data are deposited under doi:10.5061/dryad.s3h97 at the DRYAD data repository (http://datadryad.org/)

## Supporting Information

Figure S1 Further examples for sweeps and introgressed regions based on browser tracks.
(DOCX)

Protocol S1 Statistics on the effects of filtering on SNP calling.
(DOCX)

Table S1 Results of sweep statistics and candidate gene lists.
(XLS)

Table S2 Gene Ontology (GO) over-representation analysis for sweep gene lists.
(XLSX)

Table S3 Introgressed regions and Gene Ontology (GO) over-representation analysis for introgressed gene lists.
(XLSX)
