# Supplementary material for: Genome Patterns of Selection and Introgression of Haplotypes in Natural Populations of the House Mouse (Mus musculus)
Source: PLoS Genet. 2012 Aug 30;8(8):e1002891. doi: 10.1371/journal.pgen.1002891 (PMC3431316; doi:10.1371/journal.pgen.1002891)
Supplement: Figure S1 — Further examples for sweeps and introgressed regions based on browser tracks. (DOCX) [file pgen.1002891.s001.docx]

**Supplementary Figure S1**

Examples for sweep signatures and large introgressed haplotypes for the different populations (black bars on the top refer to the regions that were identified by the different statistics). The data are displayed as custom tracks in the UCSC browser. SNP positions are depicted as vertical bars, SNP variants that are more frequent in *M. m. domesticus* are in red, *M. m. musculus* in blue. Spaces between the SNP positions are filled with the color corresponding to the flanking SNPs. If these are of different color, the space is broken up in the middle. Known genes in the regions are depicted below (taken from the UCSC Genome Browser database).


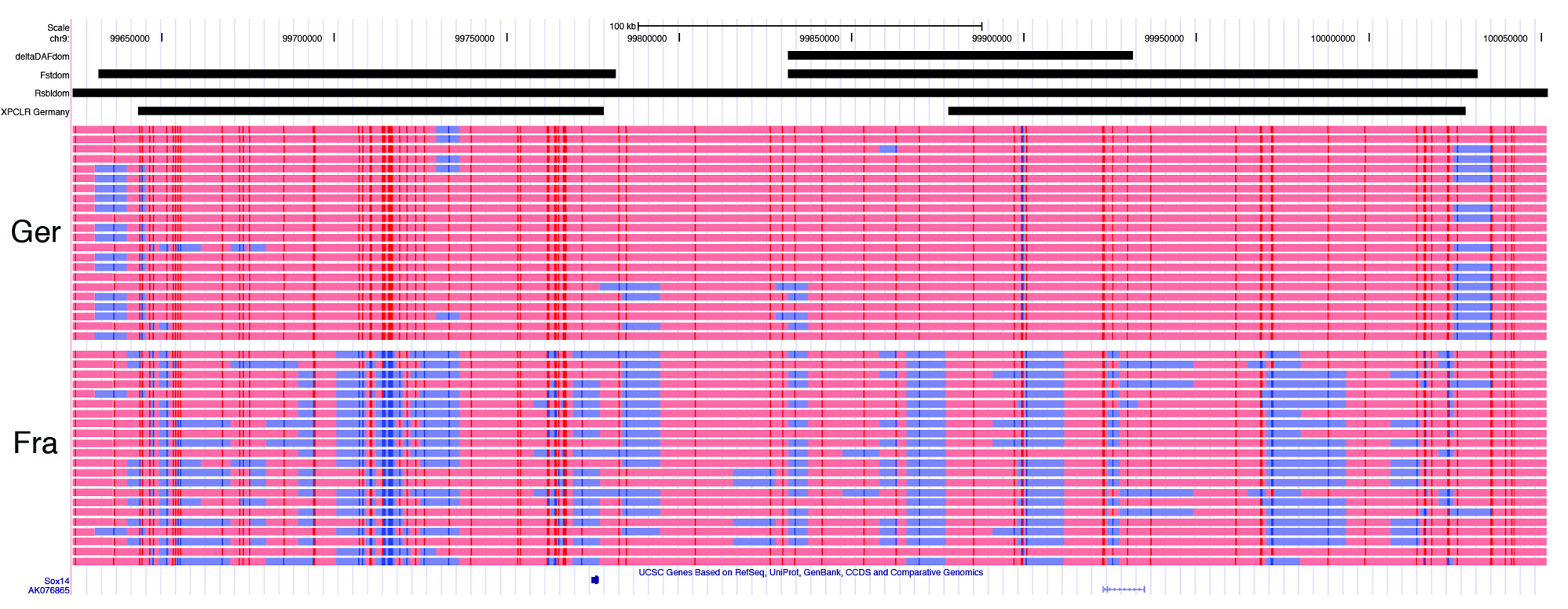


Example for a sweep in Ger. This is around the Sox14 locus on chromosome 9. It is part of a larger region identified by the different statistics, but the sweep signature is most clear in this region. Sox14 is a transcription factor that is necessary and sufficient to mediate dendrite severing during pruning in response to ecdysone signaling (Kirilly et al. 2009, Nature Neuroscience 12, 1497)


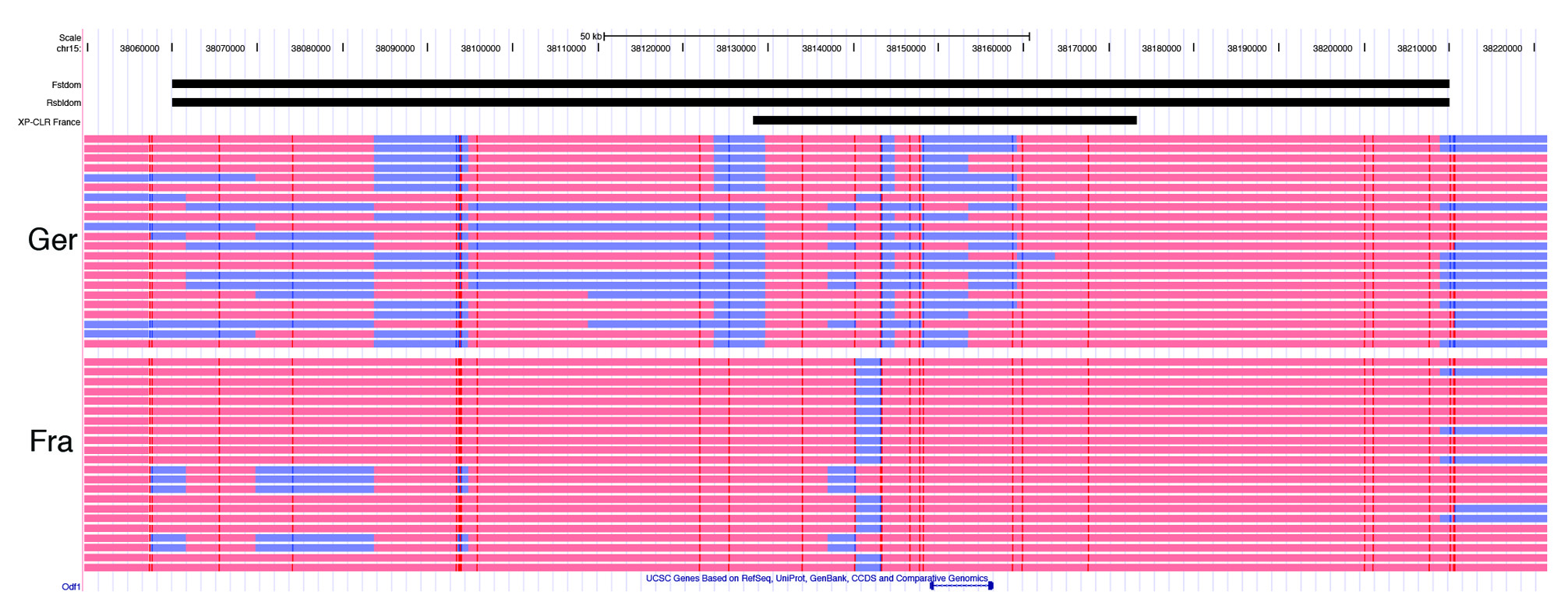


Example for a sweep in Fra. This is around the Odf1 gene on chromosome 15. Odf1 is the main protein of sperm tail outer dense fibers and belongs to the family of small heat shock proteins that function as molecular chaperones.

It is essential for tight linkage of sperm head to tail and male fertility in mice (Yang et al. 2012, Mol Cell Biol 32,

216).


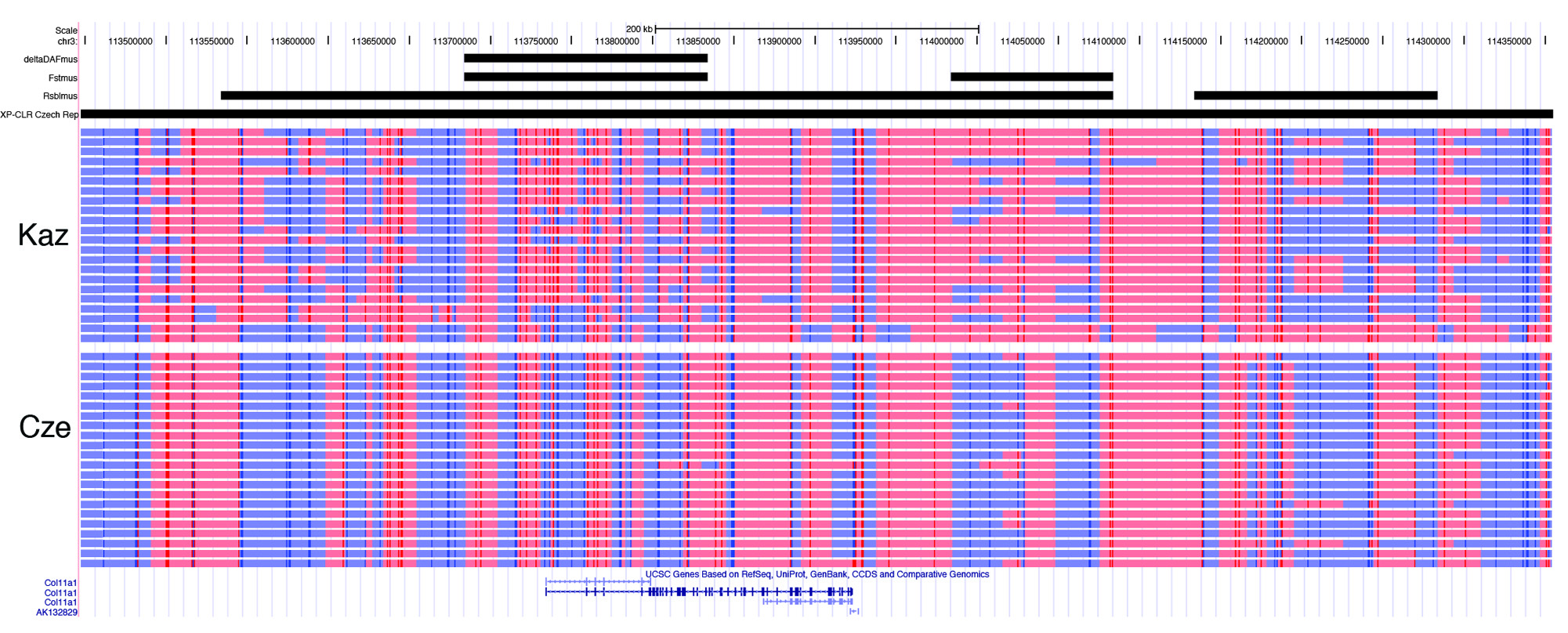


Example for a sweep in Cze. This is around the Col11a1 gene on chromosome 3. It is part of the larger region shown in Figure 7B, but here focussing on the sweep in Cze. Col11a1 is involved in craniofacial phenotypes, such as the Stickler syndrome in humans (Richards et al. 2010, Hum Mut 31, E1461)


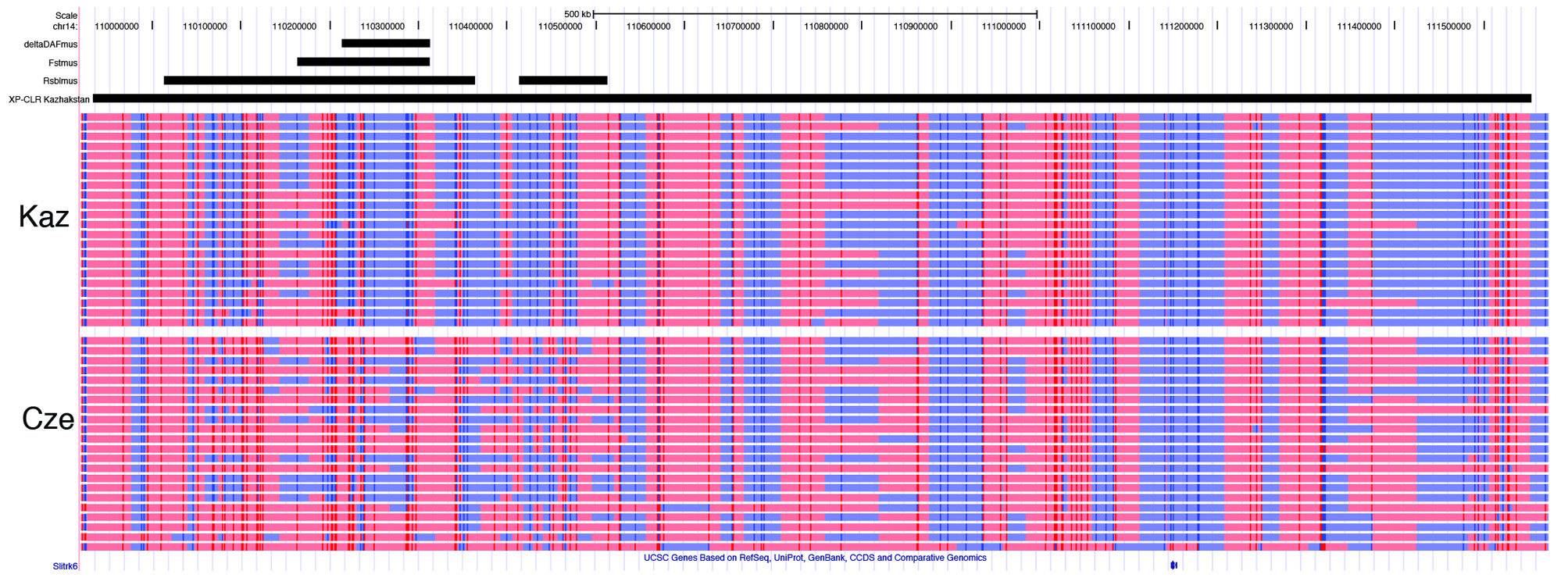


Example for a sweep in Kaz. This is around the Slitrk6 gene on chromosome 14, but mostly non-coding. Slitrk6 has a critical role in the development of the inner ear neural circuit (Matsumoto et al. 2011, PLoS ONE 6, e16497).


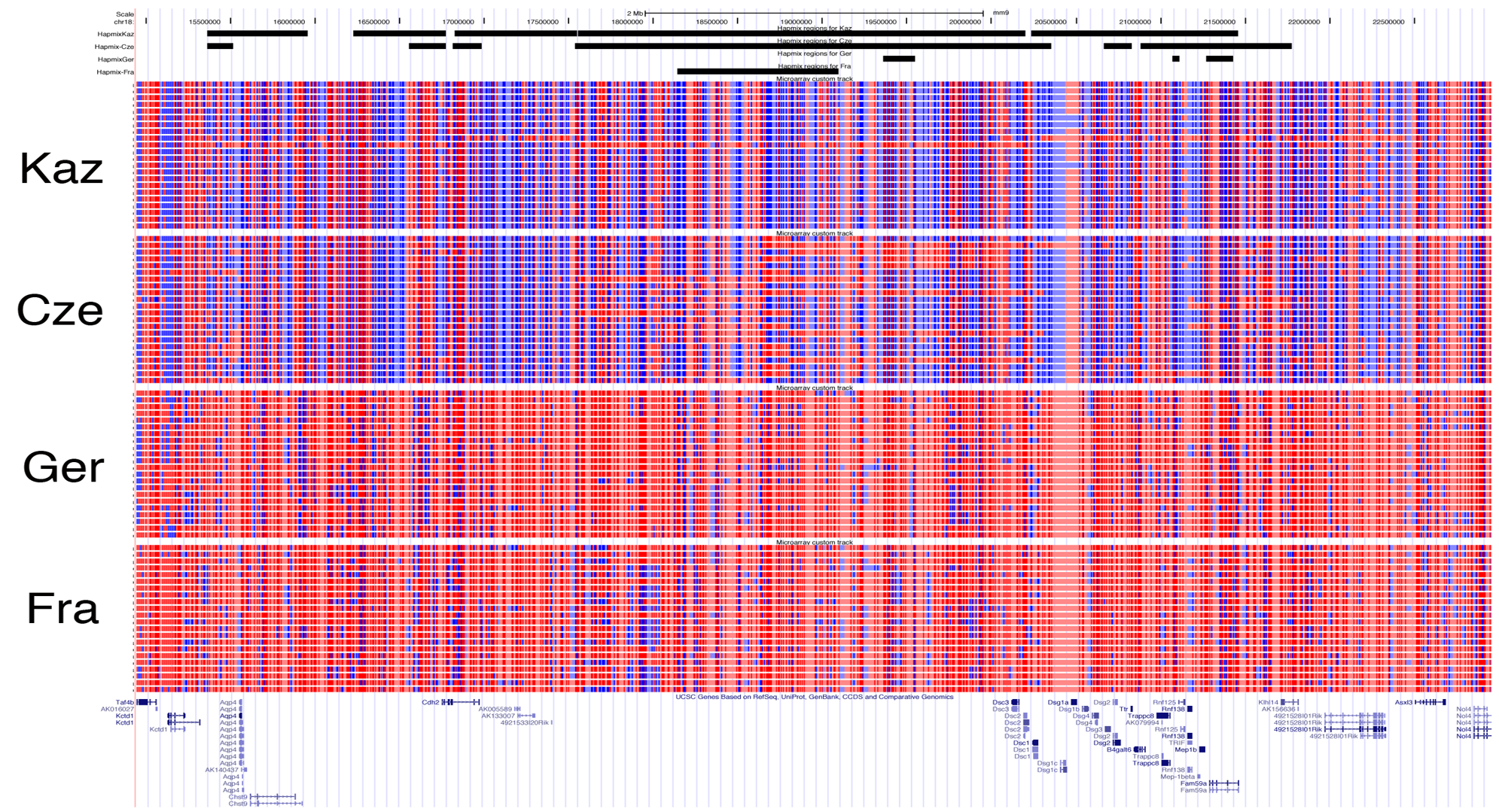


Example for a large (> 6Mb) region of complex mutual introgression, mostly in Kaz and Cze on chromosome 18. The region covers mostly a gene desert, as well as a desmocollin (dsc) and desmoglein (dsg) gene cluster, which are parts of desmosomes (intercellular junctions).


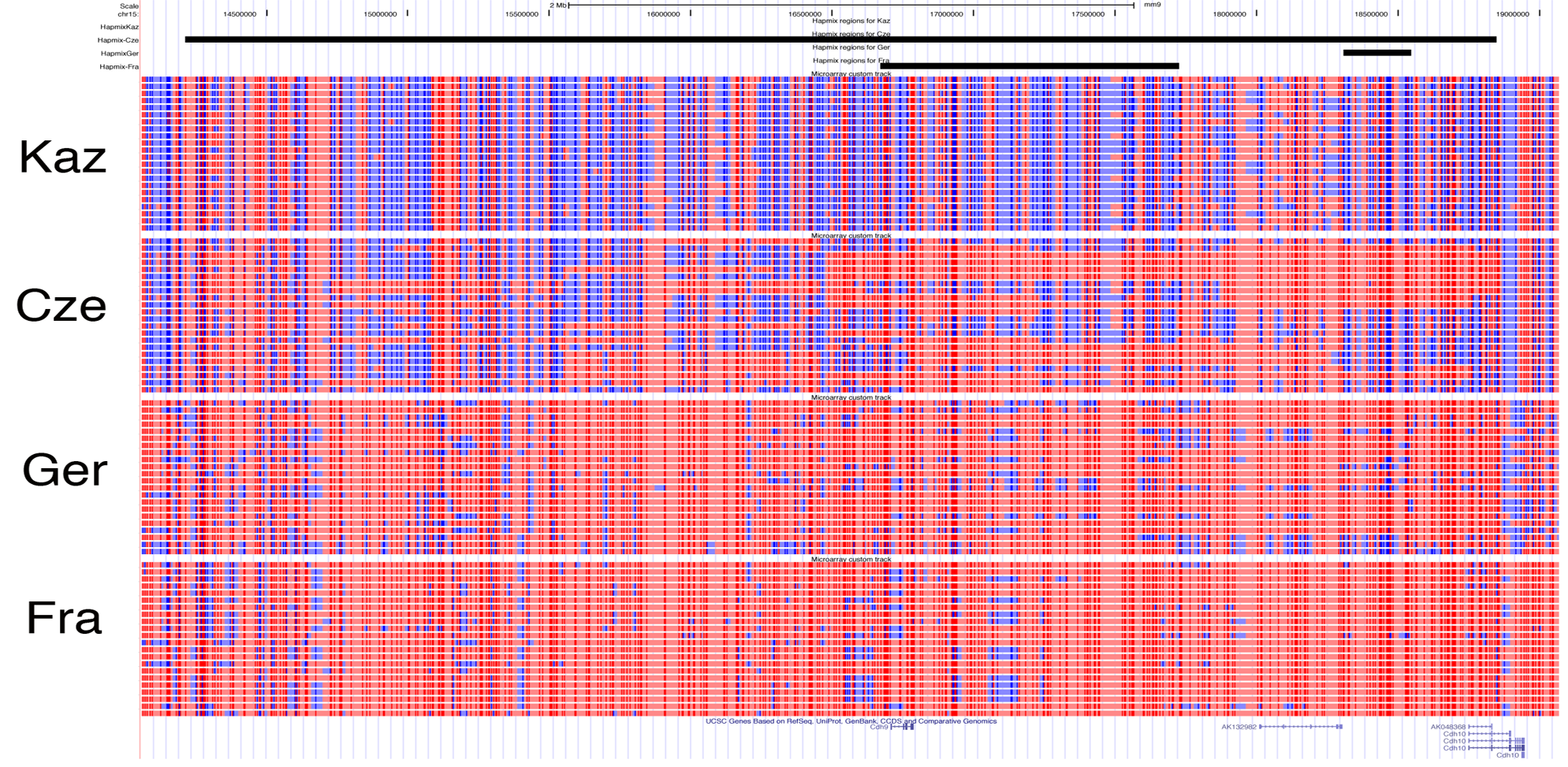


Example for a large (> 4Mb) region of complex mutual introgression, mostly in Cze on chromosome 15. The region encompasses a large gene desert surrounding the cadherin 9 gene (Cdh9).
