# Supplementary material for: Genome Patterns of Selection and Introgression of Haplotypes in Natural Populations of the House Mouse (Mus musculus)
Source: PLoS Genet. 2012 Aug 30;8(8):e1002891. doi: 10.1371/journal.pgen.1002891 (PMC3431316; doi:10.1371/journal.pgen.1002891)
Supplement: Protocol S1 — Statistics on the effects of filtering on SNP calling. (DOCX) [file pgen.1002891.s002.docx]

**Supplementary protocol S1**

**Using the MGD array with wild mice**

*by Anna Lorenc*

The Mouse Genome Diversity Array (MGDA) was designed on the basis of SNP differences between several laboratory mouse strains, both traditional and wild-derived inbred strains. When applying arrays to samples from wild *Mus musculus musculus* and *Mus musculus domesticus*, we realized that existing genotype calling methods give error rates unacceptable for downstream processing of the data with population genetics methodology, especially for mice outside the *M. m. domesticus* subspecies. Particularly, we encountered a substantial fraction of SNPs incorrectly genotyped as heterozygous.

The set of SNPs interrogated by the MGDA is biased towards SNPs present in *M. m. domesticus*, although a number of SNPs polymorphic outside of this subspecies were added. Nevertheless, for samples with increasing genetic distance to *M. m. domesticus*, probes target regions that possibly contain more and more additional polymorphisms (mismatches and indels), making one or both allele-specific probes nonfunctional.

The genotyping algorithm recommended by the array designers is Bayesian Robust Linear with Mahalanobis Distance Classifier (BRLMM-p). After normalization, probe intensities are summarized into allele signals for both alleles for each SNP and each array, S_A_ and S_B_. To assign genotypes, they are further transformed into a dimension contrasting allele intensities, for example, M = log(S_A_)-log(S_B_) and a dimension reflecting average intensity from both alleles, for example average intensity A = (log(S_A_)+log(S_B_))/2. Location of genotype clusters is estimated from these data and available as prior information. Substantially higher signal intensity (S_A_) from allele A (contrast values substantially greater than 0) lead to assigning AA genotype, higher signal intensity (S_B_) from allele B (contrast values substantially smaller than 0) lead to BB genotype, and S_A_ similar to S_B_ (contrast close to 0) – to calling AB genotype. Exact locations of cluster centers and spread of genotypes are based on prior information and estimates from the data.

As assigning genotypes is based on the contrast dimension, similar signal intensities for both alleles result in heterozygous calls. Unfortunately this is also true, when signal intensities for both alleles are very low. Very low intensity measurements for probes targeting both alleles is expected when there are sequence variants in close proximity to the targeted SNP that reduce binding affinity. Such off target variation might be frequent in natural populations and should become even more frequent with increasing phylogenetic distance between strains used for the array design and samples the array is applied to.

In our dataset, we found a fraction of SNPs called heterozygous per sample increasing with genetic distance. Also, when analyzing how many samples within a subspecies are heterozygous for a SNP, we found a surprisingly high fraction of SNPs, for which all or almost all individuals from a subspecies were called heterozygous, contrary to expectations that such cases should be extremely rare.

To estimate to what extent BRLMP is susceptible to such errors, we simulated low signal intensities from probes with additional polymorphisms, by using S_A_ values from *M. domesticus* samples genotyped as BB homozygotes and S_B_ from samples genotyped as AA homozygotes. We used highest S_A_ and S_B_ values among accessible samples. Estimates derived from maximum values might be close to real-life, as mismatches in reciprocal alleles are located in the probe center and should decrease the signal the most. Among those probesets, which should be uncalled, 67% were assigned to one of the genotypes, mostly (56 %) heterozygous. This indicates that a large fraction of non-working probes is usually incorrectly assigned with the calling software.

In the actual data, the distribution of average intensity for heterozygous calls in non-target samples suggests that misassigned genotypes are widespread. In contrast to *M. m. domesticus* samples, in *M. m. musculus* this distribution is bimodal (Figure 1) and suggests an overlap of distributions for real heterozygote genotypes and for artifact calls.


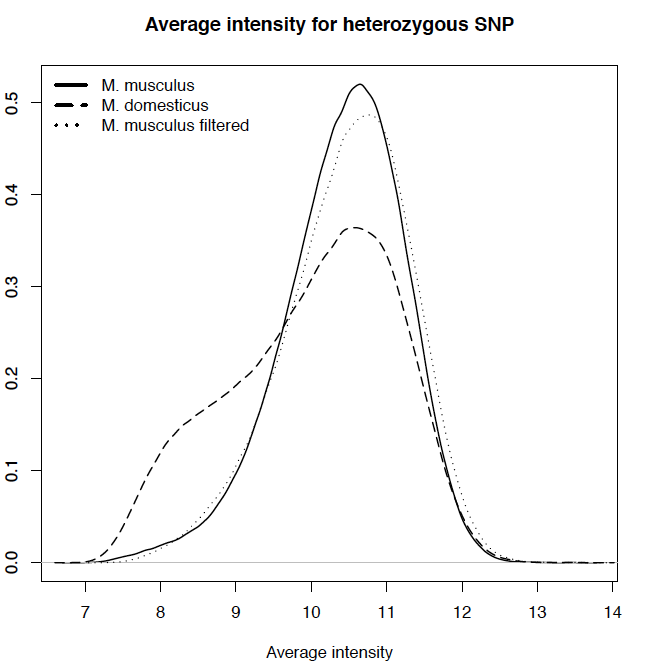


Figure 1. *M. m. musculus* average intensity for loci genotyped as heterozygous. After filtering, it is similar as for *M. m. domesticus* samples.

Furthermore, in the distribution of allele-specific probe intensities for different genotypes in *M. m. musculus*, a subset of SNPs classified as heterozygous has signal intensity similar to residual intensity in individuals lacking this allele (homozygous for another allele) (Figure 2).

| 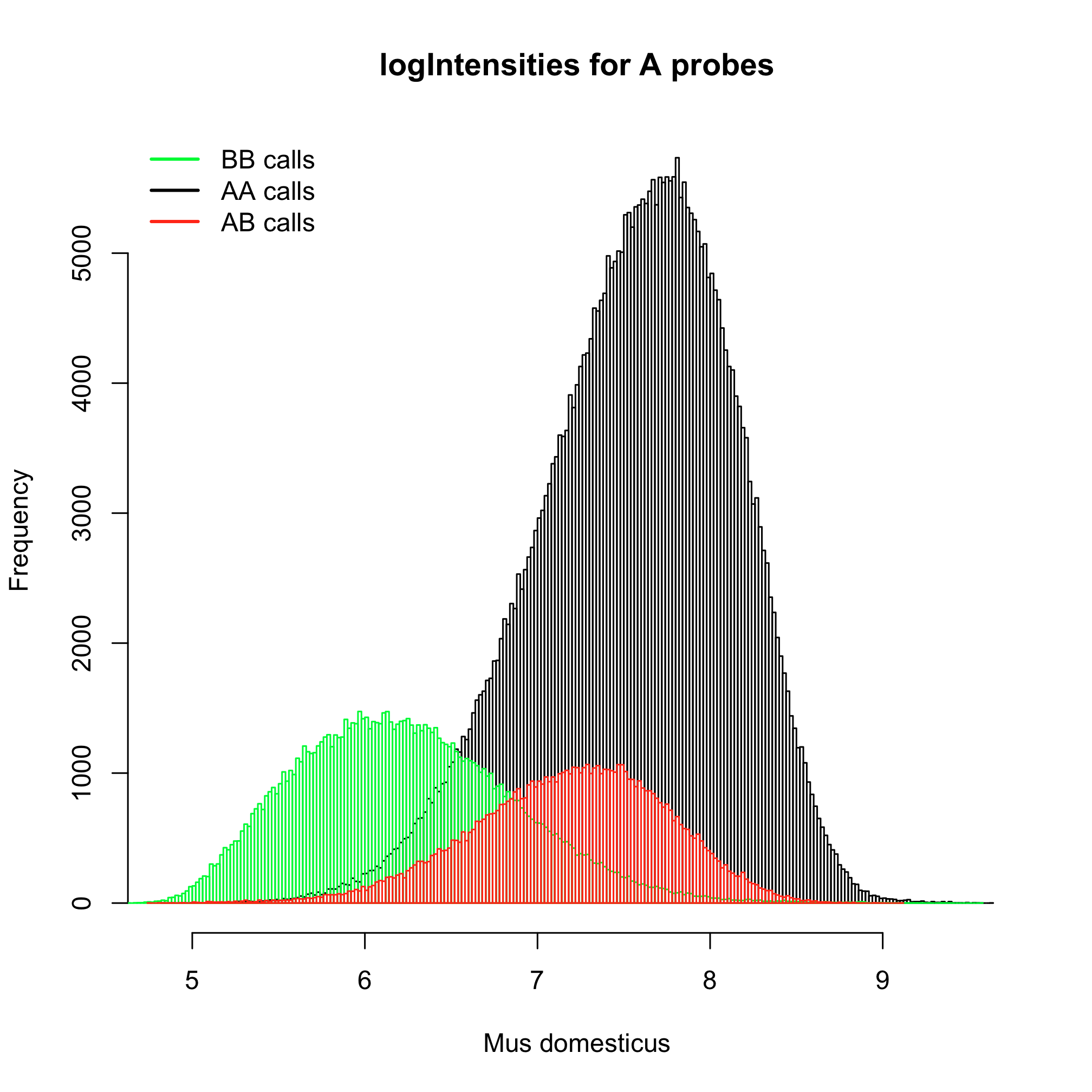 | 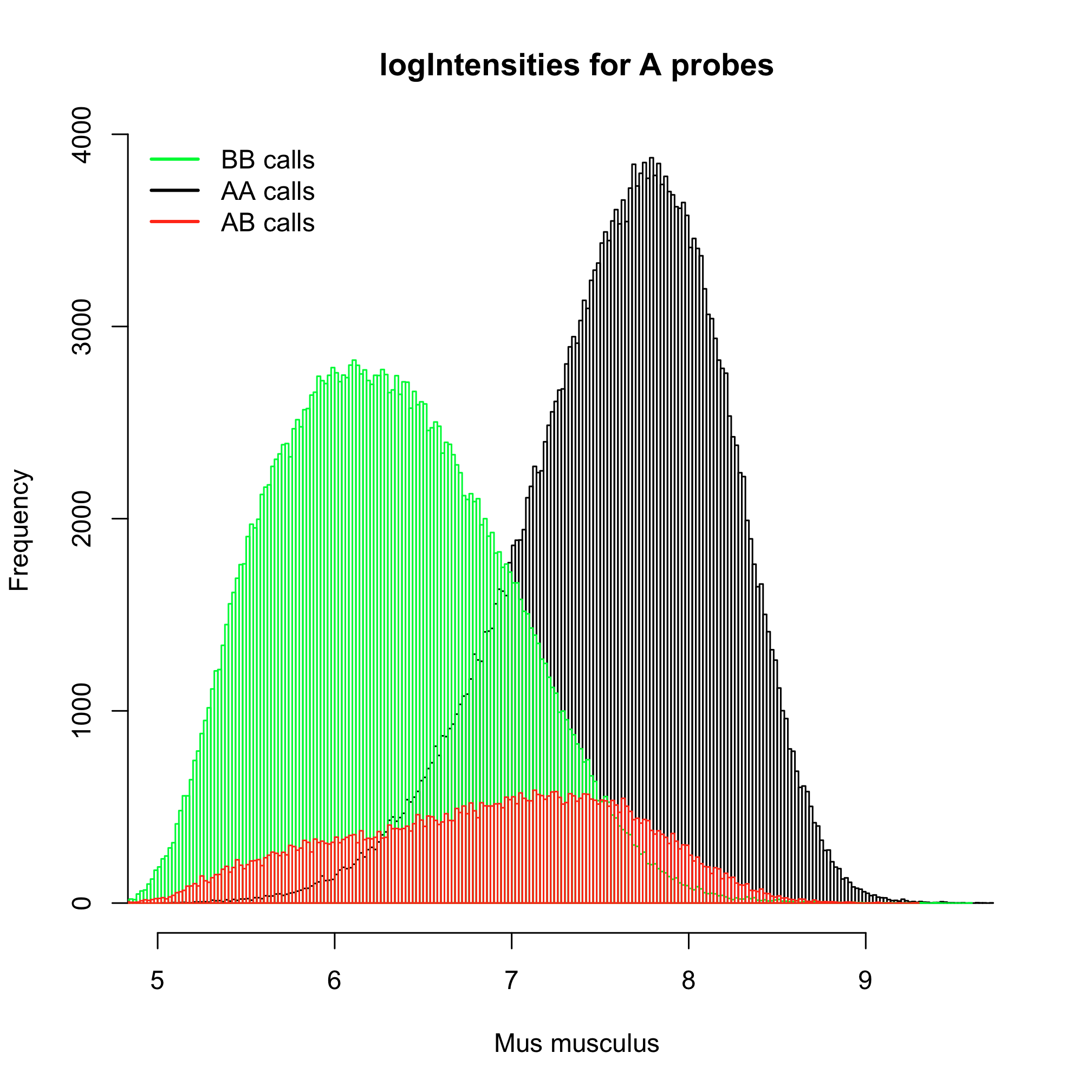 |
| --- | --- |

Figure 2: Signal from A-allele probes, plotted by genotype in comparison between *M. m. domesticus* and *M. m. musculus*.

To identify wrongly assigned heterozygous calls, we used a probeset-specific cutoff, based on reference samples. We chose to use a cutoff based on reference samples genotyped as homozygous, as it is applicable for all combinations of genotypes among reference samples, also when heterozygote calls are absent in reference samples. Moreover, it should be robust against possible incorrect heterozygous genotypes in reference samples**.** For simulated datasets, this cutoff has a detection rate 97% and average false positive rate 5%.

Applying this approach to the actual data, with 22 *M. m. domesticus* samples as a reference, we found 69,861 SNPs called as heterozygous and below the cutoff in at least one sample in *M. m. musculus*, and 100,544 of such cases in both subspecies. Treating misassigned heterozygous calls as missing reduces substantially the number of SNPs for which a high fraction of samples is heterozygous (Figure 3).

Figure 3: Fraction of SNPs called heterozygous per array. BEFORE - in raw data, AFTER - after applying our custom filter. MD - *M. m. domesticus* samples, MM - *M. m. musculus* samples. The same is shown for 3 outgroup samples (represented by single animals): *Mus caroli* (car), *Mus spretus* (spr), *Mus macedonicus* (mac) represented as single data points. The boxplots show the interquartile range with median marked and whiskers extend up to minimal and maximal values.
